# Case reports of hypercalcemia and chronic renal disease due to cosmetic injections of polymethylmethacrylate (PMMA)

**DOI:** 10.1590/2175-8239-JBN-2020-0044

**Published:** 2020-08-10

**Authors:** Arthur G. Manfro, Mauricio Lutzky, Jose M. Dora, Milton A. S. Kalil, Roberto C. Manfro

**Affiliations:** 1 Faculdade de Medicina, Universidade Federal do Rio Grande do Sul, Porto Alegre, RS, Brasil.; 2 Hospital Moinhos de Vento, Divisão de Nefrologia, Porto Alegre, RS, Brasil.; 3 Unidade de Tireoide, Divisão de Endocrinologia e Medicina Interna, Porto Alegre, RS, Brasil.; 4 Hospital de Clínicas de Porto Alegre, Divisão de Nefrologia, Porto Alegre, RS, Brasil.

**Keywords:** Polymethyl Methacrylate, Hypercalcemia, Renal Insufficiency, Polimetil Metacrilato, Hipercalcemia, Insuficiência Renal

## Abstract

Cosmetic injections of fillers are common plastic surgery procedures worldwide. Polymethylmethacrylate (PMMA) is a filler approved only for minimally invasive procedures in facial tissue and is among the most frequently used injectable substances for cosmetic purposes. Injection of a large volume of PMMA may lead to the development of severe hypercalcemia and chronic kidney damage in a probably underestimated frequency. In such cases, hypercalcemia develops due to a granulomatous foreign body reaction with extrarenal production of calcitriol. In the present report, we describe the cases of two patients who received injections of large volumes of PMMA and developed severe hypercalcemia and advanced chronic kidney disease. These reports highlight the importance of adhering to regulations regarding the use of PMMA and properly informing patients of the possibility of complications before undertaking such procedures.

## Resumo

Injeções de preenchimento de caráter estético são procedimentos comuns em cirurgia plástica em todo o mundo. O polimetilmetacrilato (PMMA) é um material de preenchimento aprovado apenas para procedimentos minimamente invasivos no tecido facial, e está entre as substâncias injetáveis mais frequentemente usadas para fins estéticos. A injeção de um grande volume de PMMA pode levar ao desenvolvimento de hipercalcemia grave e lesão renal crônica em uma frequência provavelmente subestimada. Nesses casos, a hipercalcemia se desenvolve devido a uma reação granulomatosa de corpo estranho, secundária à produção extrarenal de calcitriol. No presente artigo, descrevemos os casos de dois pacientes que receberam injeções de grandes volumes de PMMA e desenvolveram hipercalcemia grave e doença renal crônica avançada. Esses relatos destacam a importância de seguir as regulamentações sobre o uso do PMMA e informar adequadamente os pacientes sobre a possibilidade de complicações antes de realizar tais procedimentos.

## Introduction

Approximately 8.5 million cosmetic procedures using injectable fillers occurred worldwide in 2017^1^. According to the Food and Drug Administration (FDA) regulations, polymethylmethacrylate (PMMA), a semi-permanent dermal filler, is approved only for minimally invasive procedures in facial tissue around the mouth (i.e., nasolabial folds)^2^. However, an unknown frequency of the off-label cosmetic injection of this filler has been done for decades in much larger amounts in other body parts, including gluteal region and upper and lower limbs. Foreign body granulomas associated with cosmetic injections may occur in up to 1% of the cases and uncommonly may trigger calcitriol-mediated hypercalcemia, even when adequately used^3^^,^ 4. Notwithstanding, off-label injection of large volumes of PMMA can lead to the formation of significant foreign body granulomas that uncommonly lead to severe, life-threatening hypercalcemia, chronic kidney disease (CKD), and death^5^^-^ 7.

Here we report the cases of two patients that underwent aesthetic procedures with large volumes of PMMA injections who developed severe hypercalcemia and advanced CKD because of such procedures.

## Case Reports

### Case 1

A 65-year-old female sought medical attention complaining of polyuria, asthenia, and weakness in recent months. She reported recurrent urolithiasis and injections of PMMA in the face, lips, and gluteal grooves five years before. She presented in good general condition, hydrated, afebrile, with normal blood pressure and cardiac frequency. General physical examination was unremarkable and the only finding was the presence of irregular indurations on buttocks and posterior thighs.

**Figure 1:**
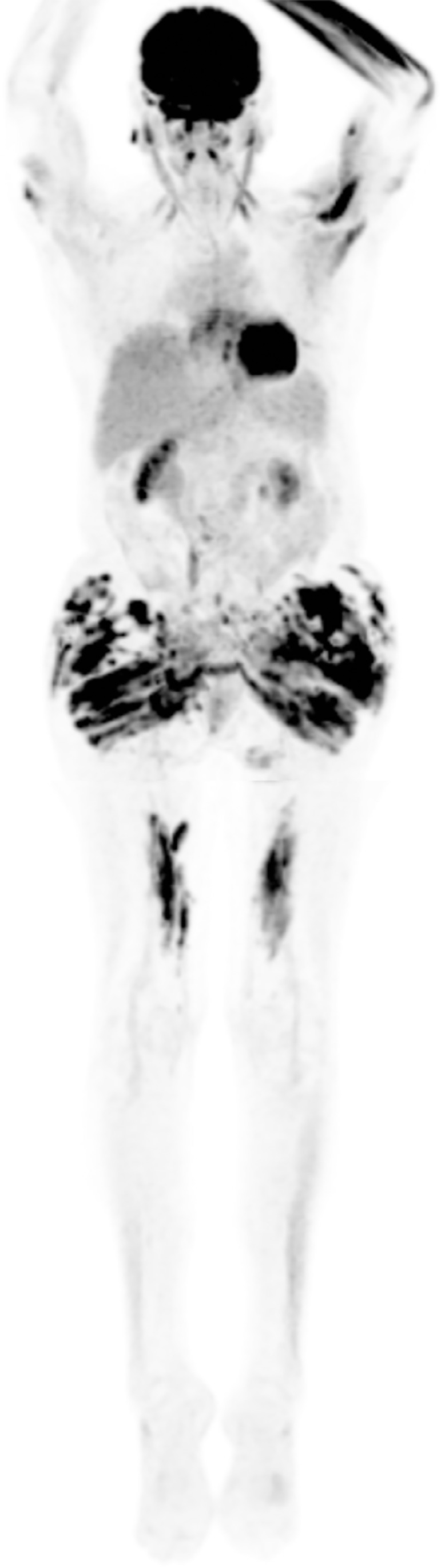
Fluorodeoxyglucose positron emission tomography (FDG-PET) demonstrating diffuse uptake in subcutaneous and muscle tissues of the gluteal region and thighs.

Laboratory evaluation showed hypercalcemia (total calcium 13.9 mg/dL, normal range [NR] 8.8 - 10.3 mg/dL), ionized calcium 1.98 mg/dL, NR 1.1 - 1.35 mmol/L) increased serum creatinine (1.7 mg/dL, MDRD estimated glomerular filtration rate [eGFR] 31 mL/min/1.73m^2^), and increased blood urea nitrogen (45 mg/dL), with normal blood range levels of sodium, potassium, bicarbonate, uric acid, albumin, and phosphorus. Twenty-four-hour urine analysis revealed mild proteinuria (650 mg/24h) and hypercalciuria (350 mg/24h), with normal uricosuria, citraturia, phosphaturia, and oxaluria. Urinalysis presented ++/4 proteinuria, and urine was sterile in culture. Abdominal ultrasound and computerized tomography showed multiple bilateral urolithiasis. Intact PTH (46 pg/mL, NR 15-65 pg/mL) and 25(OH) vitamin D3 (22 ng/mL, NR 20 - 60 ng/mL) levels were within normal ranges while 1-25 (OH)_2_D_3_ (84 pg/mL, NR 16.0 - 65.0 pg/mL) was increased. Intravenous saline hydration and prednisone (60 mg QD) were started for the management of hypercalcemia attributed to a granulomatous reaction due to the PMMA implants. A fluorodeoxyglucose positron-emission tomography (FDG-PET) demonstrated diffuse fluorodeoxyglucose uptake in subcutaneous and muscle tissues of the gluteal region and thighs (Figure 1). The surgical consultation ruled out the possibility of removing the PMMA implants. The patient refused therapy with bisphosphonates given the risk of mandibular osteonecrosis.

Hypercalcemia persisted and the renal function deteriorated (serum creatinine of 6.1 mg/dL, MDRD eGFR 6 mL/min/1.73m^2^). The patient became uremic and underwent intermittent hemodialysis with low dialysate calcium (2.5 mEq/L) for a brief period. At this time she received denosumab with partial response of the serum calcium (10.7 mg/dL) and renal function, both not sustained, leading to denosumab discontinuation. Over the following two years, hypercalcemia persisted and the renal function kept deteriorating, eventually requiring chronic renal replacement therapy with hemodialysis.

### Case 2

A 69-year-old female patient was admitted to the hospital complaining of asthenia, malaise, and weight-loss. The patient had no relevant previous medical history and no family history for kidney disorders. She reported having performed injections of PMMA gel on both arms eight months before admission. She presented pale, in good general condition, normal blood pressure, cardiac rate, and temperature. There was no abnormality in physical examination, except for the presence of dense nodules in forearms.

Laboratory findings were anemia, increased serum creatinine (2.6 mg/dL, MDRD eGFR 18 mL/min/1.73m^2^), normal urinalysis, and absence of proteinuria. Renal ultrasound showed normal-sized kidneys. The attending team suspected of vasculitis and a renal biopsy was performed and a methylprednisolone pulse was initiated. The renal function further declined (creatinine 3.43 mg/dL, MDRD eGFR 13 mL/min/1.73m^2^) and hypercalcemia (ionized calcium 1.54 mmol/L) was detected. Laboratory work up revealed normal serum protein electrophoresis, ANCA, anti-cardiolipin, lupus anticoagulant, ANA, serum complement levels, anti-DNA, anti-HIV, anti-HCV, and HBsAg. Serum levels of intact PTH were 25.3 pg/mL, 25(OH) vitamin D3 42ng/mL, and 1-25 (OH)_2_D_3_ 52.3 pg/dL. Biopsy revealed normal glomeruli, tubular degeneration with atrophy, moderate interstitial fibrosis, and discrete arterial intimal hyperplasia, but no vasculitis. Prednisone was tapered to 20 mg QD and kept for management of hypercalcemia secondary to granulomatous reaction due to PMMA implants. Therapy succeeded in lowering calcium (serum ionized calcium 1.2 mmol/L) and improving renal function (serum creatinine 1.3 mg/dL, MDRD eGFR 41 mL/min/1.73m^2^).

Due to the cosmetic effects, the patient discontinued the steroid treatment returning after four years with a loss of renal function (serum creatinine 2.86 mg/dL, MDRD eGFR 16 mL/min/1.73m^2^), and hypercalcemia (serum total calcium 11.0 mg/dL). Reintroduction of prednisone led to an improvement in renal function and hypercalcemia. However, the patient persisted with the pattern of poor adherence, with recrudescence of hypercalcemia and worsening chronic kidney disease. The surgical consultation team performed the removal of the larger granulomas of the right forearm but several implants could not be removed and no response in hypercalcemia occurred. Histological examination showed deposits of inorganic globular material in fibro adipose tissue surrounding muscle fasciculi leading to granulomatous foreign body reaction. Currently, the patient is on prednisone (10 mg QD), with controlled serum total calcium (9.7 mg/dL) and receiving conservative treatment for stage IV chronic kidney disease (MDRD eGFR 22 mL/min/1.73m^2^).

Table 1 summarizes the main clinical and laboratory characteristics of the two cases.

**Table 1:** Main clinical and laboratory characteristics of patients with hypercalcemia and renal damage that received esthetic injections of polymethylmethacrylate.

Patient	Age	Gender	Symptoms	Injection site	Serum calcium*	Serum creatinine*	Treatments	Outcome
**1**	65	Female	Polyuria, asthenia, weakness	Buttocks	13.9 mg/dL (total)	1.7 mg/dL	Steroids, Denozumab	CKD stage 5 Hemodialysis
**2**	69	Female	Asthenia, malaise, weight loss	Arms	1.54 mmol/L (ionized)	2.6 mg/dL	Steroids, surgical removal	CKD stage 4

* At presentation

## Discussion

In this report, we described two cases of severe hypercalcemia and CKD due to granulomatous reactions to PMMA injections used in aesthetic filling procedures. Although uncommon, granulomatous reactions may occur as a response to the presence of permanent filling materials such as silicones, PMMA, and paraffin. Kozeny and collaborators originally described the occurrence of hypercalcemia with the use of silicone injections^8^. Later, a case series of four patients called attention to the occurrence of hypercalcemia and CKD in patients that received PMMA in the gluteus and lower limbs^5^. In one of their cases, a muscle biopsy showed PMMA globules surrounded by a granulomatous inflammatory reaction, describing the pathological process related to hypercalcemia in such cases. More recently, Hindi and collaborators demonstrated the overexpression of CYP27B1 in skeletal muscle lesions of an HIV infected patient that developed a granulomatous reaction five years after injecting PMMA. The enzyme CYP27B1 seems to be highly specific for catalyzing 1α-hydroxylation on a range of endogenously produced vitamin D metabolites and its overexpression supports extra-renal calcitriol production, by activated macrophages, in a foreign body reaction to PMMA, as a mechanism for hypercalcemia^9^^,^ 10.

Both patients presented with severe hypercalcemia and loss of renal function detected over the years (case 1) or months (case 2) after an injection of a large volume of PMMA. The described cases, despite similarities in clinical and laboratory presentation, had distinct diagnostic approaches due to initial clinical hypothesis of the attending teams. In the first case, the presence of urolithiasis incited an anticipated evaluation of calcium levels; in the second case, however, this investigation was made in a second moment, after the worsening of kidney function despite treatment for suspected vasculitis. Interestingly, both patients presented with normal, non-suppressed PTH, in spite of very elevated serum calcium, normal levels of 25-hydroxyvitamin D, and increased level of 1,25(OH)_2_D_3_ in one case. In our first patient, the hypercalcemia response to corticosteroids and denosumab was not sustained, and the renal function deteriorated. However, partial and transitory recovery of renal function occurred after near normalization of the serum calcium with hemodialysis with low dialysate calcium, suggesting that the renal failure was partially mediated by hypercalcemia-induced arteriolar vasoconstriction^(11, 12)^. The second patient presented a much better response to corticosteroid therapy, however, the steroid-related cosmetic effects led her to a non-adherence behavior and recrudescence of hypercalcemia, which led to a deterioration of the renal function. The reasons why one patient responded to corticosteroid therapy and the other did not are not clear. We can only hypothesize that the amount of PMMA inoculum and perhaps the possibility of partial removal in one of the cases might be involved in the outcome.

Recently, Tachamo and collaborators published a systematic review of 23 cases of patients with hypercalcemia associated with cosmetic injections. Silicone was the most used filler, followed by PMMA. The vast majority of patients were females (cisgender and transgender), the most common body parts for injection were buttocks, breasts, and thighs. In this report, hypercalcemia was discovered on average eight years after the cosmetic procedures. The majority of the patients presented renal failure as a complication of hypercalcemia and two patients died. Elevated calcitriol level was present in two-thirds of the patients and suppression of the PTH occurred in more than 80% of the cases. Recurrence of hypercalcemia occurred in almost half of the patients despite a variety of treatments^7^.

Our cases share similarities and differences with the main behavior of the cases described by Tachano et al^7^. Gender, hypercalcemia-related symptoms at onset, and, most importantly, occurrence of significant and permanent loss of renal function were seen as the main similarities. However, our patients were older, presented non-suppressed PTH, and one of them did not have elevated calcitriol levels. Perhaps, the non-suppressed PTH can be at least partially explained by the occurrence of secondary hyperparathyroidism. Normal range calcitriol levels were also found in one-third of the patients in a previous systematic review, therefore, not excluding hypercalcemia related to granulomatous reaction. Reasons why calcitriol levels can be normal in such patients are yet to be understood, but they might be related to baseline 25-hydroxyvitamin D levels^7^. However, the ultimate reasons for the differences are not possible to uncover at present. Moreover, one must consider that the Tachamo systematic review included patients that received different implants other than PMMA. It is conceivable that these fillers may produce different biological reactions leading to distinct laboratory phenotypes.

Briefly, the mechanism of renal damage is related to severe long-standing hypercalcemia and hypercalciuria, which may lead to the development of interstitial fibrosis and nephrocalcinosis causing CKD. In such patients, the usual clinical presentations are polyuria, nephrolithiasis, type 1 renal tubular acidosis, and renal failure. The intensity and chronicity of the kidney damage are dependent on hypercalcemia severity and duration^11^^,^ 13.

In conclusion, we recommend following the regulations regarding the use of PMMA and believe that there is enough evidence to recommend warning patients of the uncommon but noxious complications associated with such procedures. A multidisciplinary collaboration may help uncover the actual frequency and relevant aspects of hypercalcemia and renal damage associated with the use of PMMA fillers.
